# Supplementary material for: Combined analysis of microbial metagenomic and metatranscriptomic sequencing data to assess in situ physiological conditions in the premature infant gut
Source: PLoS One. 2020 Mar 4;15(3):e0229537. doi: 10.1371/journal.pone.0229537 (PMC7055874; doi:10.1371/journal.pone.0229537)
Supplement: S1 File — Fig A. Relative abundances of different Bacterial genera; Table A. Accession phrases for Escherichia spp. genes in on ggkbase interface; Table B. Percent of shared affiliation of the predicted proteins. (DOCX) [file pone.0229537.s001.docx]

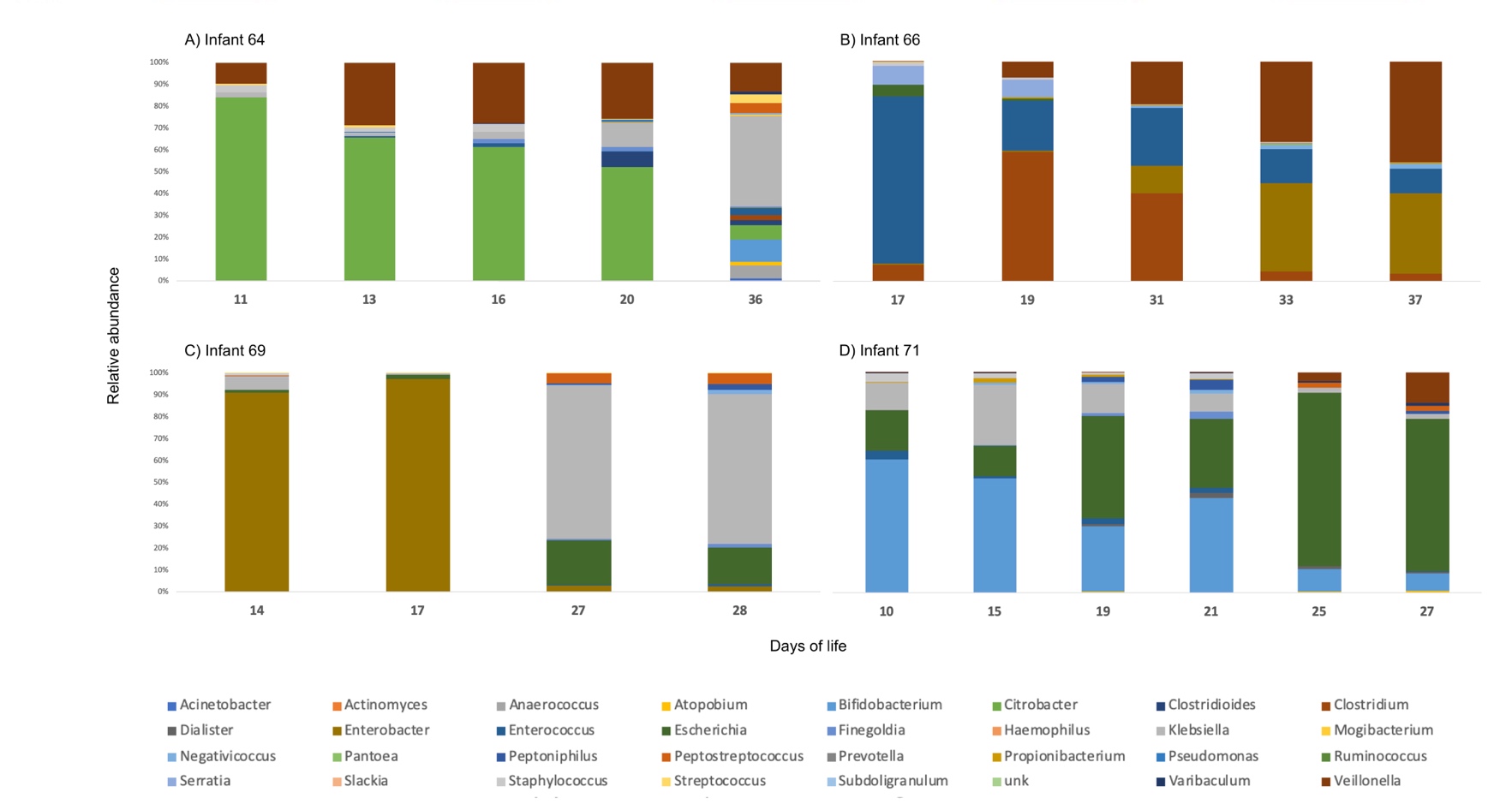


**Fig A. Relative abundances of different Bacterial genera found in stool samples of premature infants on different days of life. Infants 64 and 66 are control infants, infants 69 and 71 were later on diagnosed with Necrotizing enterocolitis.**

**Table A. Accession phrases for Escherichia spp. genes in on ggkbase interface (**[**https://ggkbase.berkeley.edu/**](https://ggkbase.berkeley.edu/)**) used in this study**

| **Coupled genes for DR** | **Genes** | **Scaffolds used for the different infants** | | | |
| --- | --- | --- | --- | --- | --- |
|  |  | **Infant 71** | **Infant 69** | **Infant 66** | **Infant 64** |
| **1)** | ***cydAB*** | 71_013_scaffold_12_143-144 | 69_008_scaffold_426_11-10 | 66_004_scaffold_7_85-84 | N2_064_000G1_scaffold_3505_6-5 |
| **1)** | ***cyoABCD*** | 71_013_scaffold_3_97-100 | 69_008_scaffold_38_77-74 | 66_004_scaffold_23_112-115 | N2_064_000G1_scaffold_1959_5-8 |
| **2)** | ***arcA*** | 71_013_scaffold_7_194 | 69_008_scaffold_6_24 | 66_004_scaffold_32_26 | N2_064_000G1_scaffold_499_1 |
| **2)** | ***fnr*** | 71_013_scaffold_10_62 | 69_008_scaffold_39_24 | 66_004_scaffold_22_279 | N2_064_000G1_scaffold_1695_1 |
| **3)** | ***nrdDG*** | 71_013_scaffold_7_318-19 | 69_008_scaffold_6_178-179 | 66_004_scaffold_8_200-199 | N2_064_000G1_scaffold_3_430-431 |
| **3)** | ***nrdAB*** | 71_013_scaffold_13_152-151 | 69_008_scaffold_35_2-3 | 66_004_scaffold_6_87-86 | N2_064_000G1_scaffold_327_42-41 |
| **4)** | ***norVW*** | 71_013_scaffold_6_83-84 | 69_008_scaffold_67_16-17 | 66_004_scaffold_66_52-53 | N2_064_000G1_scaffold_620_14-15 |
| **4)** | ***norR*** | 71_013_scaffold_6_82 | 69_008_scaffold_67_15 | 66_004_scaffold_66_54 | N2_064_000G1_scaffold_620_13 |
| **5)** | ***ompC*** | 71_013_scaffold_13_171 | 69_008_scaffold_1_16 | 66_004_scaffold_6_93 | N2_064_000G1_scaffold_844_3 |
| **5)** | ***ompF*** | 71_013_scaffold_17_90 | 69_008_scaffold_116_19 | 66_004_scaffold_11_234 | N2_064_000G1_scaffold_923_6 |

**Table B. Percent of shared affiliation of the predicted proteins on the same scaffold of each of Escherichia spp. genomes**

|  | **Super-kingdom** | | **Phylum** | | **Class** | | **Order** | | **Family** | | **Genus** | | **Species** | |
| --- | --- | --- | --- | --- | --- | --- | --- | --- | --- | --- | --- | --- | --- | --- |
| **Infant** | **%** | **Winner** | **%** | **Winner** | **%** | **Winner** | **%** | **Winner** | **%** | **Winner** | **%** | **Winner** | **%** | **Winner** |
| **64** | 100 | Bacteria | 95 | Proteobacteria | 95 | Gammaproteobacteria | 95 | Enterobacterales | 94 | Enterobacteriaceae | 90 | Escherichia | 90 | E. coli |
| **66** | 100 | Bacteria | 99 | Proteobacteria | 99 | Gammaproteobacteria | 99 | Enterobacterales | 97 | Enterobacteriaceae | 85 | Escherichia | 84 | E. vulneris |
| **69** | 100 | Bacteria | 100 | Proteobacteria | 100 | Gammaproteobacteria | 100 | Enterobacterales | 99 | Enterobacteriaceae | 97 | Escherichia | 96 | E. coli |
| **71** | 100 | Bacteria | 100 | Proteobacteria | 100 | Gammaproteobacteria | 100 | Enterobacterales | 100 | Enterobacteriaceae | 96 | Escherichia | 95 | E. coli |
