# Supplementary material for: Combined analysis of microbial metagenomic and metatranscriptomic sequencing data to assess in situ physiological conditions in the premature infant gut
Source: PLoS One. 2020 Mar 4;15(3):e0229537. doi: 10.1371/journal.pone.0229537 (PMC7055874; doi:10.1371/journal.pone.0229537)
Supplement: Attachment — Submitted filename: Response to Reviewers_v2.docx [file pone.0229537.s009.docx]

**Response to Review Comments**

**Reviewer #1**:

Yonathan Sher et al describe a method to study physiological conditions based on bacterial gene expression, applied on feces derived from preterm infants that do or don’t develop NEC. While the approach in itself is interesting and could be applied in multiple settings, the method is not described in enough detail to be repeated by others, it is not validated under known ‘physiological conditions’ and I doubt its applicability in diagnostic setting as proposed by the authors. Since NEC most often occurs after 2 to 4 weeks of life, time is a limiting factor when using such methodology for early NEC diagnosis. See my detailed comments below.

***Response:*** *Thank you for these comments.*

*We have made many revisions according to both reviewer’s comments, and we hope now this reviewer will agree that the methods are detailed enough for this approach to be repeated by others, specifically the section describing diametric ratio formula and the rationale behind it. (Lines 143-160)*

*Previous knowledge in the literature, from biochemical studies on isolates, direct our choice of genes for calculating diametric ratio and constitute a validation for transcriptional response to the physiological conditions. In addition, to add more rigor to the response to oxygen level, several diametric genes sets were examined (cydAB/cyoABCD; nrdGD/nrdAB; fnr/arcA).*

*We do agree that further research is needed to fully validate the ability of RNA-seq to recapitulate physiological conditions in the gut, potentially involving experiments where infant feces are inoculated into artificial media with known physiological condition and in experiments with animal models inducing the relevant physiological conditions. (Lines 364-367), however we believe these extensive validations are outside the scope of the current bioinformatic study.*

*The method by is not a diagnostic method, nor to we claim it to be one. We clearly mention this fact in the discussion section and have moved these lines to end of the text to be more noticeable (Lines 394-396). We have also added a line in the end of the abstract to be clear that this approach is a useful research approach, from which diagnostic tools can be designed.*

[General]
- Many grammatical and spelling errors throughout the manuscript

***Response:*** *Thanks for the comment. Additional proof reading was done by professional native English speakers to reduce grammatical and spelling errors.*

- Is oxygen deprivation in the gut of preterm infants a local issue, or does it affect the complete intestine? Does it affect both the small and large intestine? What was the situation for the two included NEC infants? If affecting the small intestine in a local manner, are feces samples a good representative?

***Response:*** *The comment is insightful but it is not possible to fully answer the questions with currently available data.*

*Oxygen deprivation in the gut can be local or diffuse, but NEC is generally considered a disorder of system-wide inflammation. Oxygen availability in the intestine, which varies between the lumen and the mucosal surface, is multifactorial. It appears to be determined by blood flow (and hemoglobin concentration/oxygen carrying capacity), the presence of inflammatory cells, as well as mitochondrial activity of gut epithelial cells which utilize locally produced oxygen species to generate ATP.*

*Inflammation associated with NEC can be found in either small and large intestine or both (Lines 52-54; Lines 296-297). The diagnosis of NEC is often made with clinical and radiographic criteria without detailed knowledge of the precise location of disease. One of the two infants with NEC studied here died suddenly from fulminant disease prior to surgical intervention. Because surgery was not done– which would enable a detailed characterization of the regional distribution of disease – we cannot say exactly whether the disease involved both the small and/or large intestine. The second infant indeed did undergo surgery for advanced NEC. We have re-reviewed our study notes from that time several years ago, but unfortunately the location of disease identified at surgery was not recorded in our notes. We do not have IRB approval to look back in the patient record to discern this point.*

*As the reviewer suggests, it is possible that fecal samples are not adequate for assessing the presence/absence of inflammation in the small intestine. However, it is also certainly possible that fecal samples ARE adequate. More samples and more infants must be studied to make this determination.*

*However, as stool flow pretty fast through the intestine of preterm infants (as evident by high stool frequency of between 2-5 stools a day- see Weaver and Lucas, 1993), we believe that feces samples are a good representative.*

*Weaver and Lucas (1993) Archives of Disease in Childhood, 68:317-320.*

[Introduction]
Line 36: remove dash for in-vivo.

***Response:*** *Thank you, the dash has been removed. (Line 42)*

Line 64: ‘can be used to the study’ change to ‘can be used to study’.

***Response:*** *Thank you, changed. (Line 70)*

[Methods]
Used software is without version numbers and parameters/settings and can therefore not be reproduced by others.

***Response:*** *Version numbers were added. Unless stated, default settings were used. (Line 99-109)*

Lines 69-71: I encourage a more detailed description of how fecal samples were processed and stored to indicate compatibility for RNA sequencing. In addition, the manuscripts focusses on oxygen exposure, which may be challenging taking the method of fecal sample collection into consideration.

***Response:*** *As noted, samples were collected as soon as possible after evacuation and stored at -80^o^C until DNA and RNA extraction (Line 75-84). The samples were collected as part of a large sample collection campaign, which was led by a single research coordinator in close communication with NICU nurses, thus minimizing variability in sample collection. Samples were generally collected soon after evacuation, and not more than 3 hours after evacuation. The samples were collected in standard fecal sample collection tubes without additional preservative. This method was because preliminary experiments (not shown) with and without RNA preservative demonstrated superior RNA quality with this approach. RNA quality was verified for all samples that underwent transcriptomic sequencing.*

Lines 88: Change ‘which use’ to ‘which uses’.

***Response:*** *Thank you, changed. (Line 101)*

Lines: 92-95: Does this mean these scaffolds are not yet made available? Please make them available with the next submission.

***Response:***  *Are available with next submission (Line 103)*

Line 99: Change ‘phyla level’ to ‘phylum level’.

***Response:*** *This text was removed altogether.*

Line 103: Write HMMs fully, since it is the first and only time the word is used.

***Response:*** *Thank you, changed. (Line 128)*

Line 109-110: Please clarify what is filtered for by mapped.py. How does this complement to the mapping performed using Bowtie2?

***Response:*** *This script filters out any reads that map with more than one mismatch to the reference genome. This stringent filtering was used to increase our confidence that RNA reads are mapped to the correct genome. To clarify what this script does we added in the following sentence in the text:*

*“This script filters out any reads that map with more than one mismatch to the reference genome.” (Lines 136-137)*

Line 112: Use of "coverage" is ambiguous: breadth or depth? (see also <https://www.danielecook.com/calculate-depth-and-breadth-of-coverage-from-a-bam-file/>)

***Response:*** *We used the values of coverage depth. To resolve any ambiguities, we modified the term "coverage"* *to "coverage depth". (Line 139-140)*

Line 116-120: This equation only takes transcript abundances into account. I wonder how this corrects for genome abundance. Did the authors considered to correct for genome abundance by using their metagenomics data? Or by other means (by correcting for total reads or total mapped reads (https://www.biostars.org/p/273537/). Also, please clarify how opposite regulating genes are identified. What does opposite regulating genes mean exactly and how can one find them for their function of interest?

***Response:*** *Thank you for this comment. The rationale behind the diametric ratios is to examine transcriptional responses of specific genes, and at the same time avoid biases related to differences in genome abundances. When constructing the diametric ratios from transcripts of genes from the same genome it eliminates biases due to genome abundances, as within the diametric ratio formula genome abundances factor out. To better explain this concept, we rewrote the text describing diametric ratios. In addition, the term ‘regulating genes’ has been replaced with the term ‘opposite transcriptional response’. The method section has been re-written as follows:*

*“Gene expression levels were measured using transcript ratios, calculated according to equation (*a*):*

$$\left( \text{a} \right) DR\left( G, E \right)=\frac{\overline{RNA\left( \alpha_{G} \right)}}{\overline{RNA\left( \alpha_{G} \right)}+\overline{RNA\left( \beta_{G} \right)}} \begin{matrix} where \alpha are genes within G that increase in response to E \\ and \beta are genes within G that decrease in response to E \end{matrix}$$

*Where α is the transcript (RNA) abundance of a certain gene/genes (average abundances if abundances of several genes are considered, as denoted with the overbar) in a specific genome (G), and β is the transcript (RNA) abundance of a different gene/genes in the same genome (G) with an opposite transcriptional response to a surrounding physiological condition (E).*

*For example, if a certain surrounding physiological condition (E; e.g. oxygen level) increases transcription of gene α in genome G than for the same physiological condition (E) transcription of gene β in genome G decreases (Table 3). Because of the opposite transcriptional responses of examined genes these transcript ratios are referred to as diametric ratios (DR). Calculating transcriptional responses this way allows measuring shifts in transcriptional responses while minimizing biases associated with changes in genome abundance. As noted, transcripts of the two sets of genes (α and β) originate from the same genome (G). Thus, given that the values of α and β are composed from transcript abundance per genome multiplied by genome abundance, the DR formula factors out genome abundance and the DR are comparable between samples. “ (Lines 143-160)*

*Genes with opposite transcriptional response were chosen a-priori according to their function description in the literature. This is discussed in the discussion section. (Lines 370-371)*

[Results]
Write the section in past tense

***Response:*** *Where needed the text was change to past tense*

Line 140: Change to ‘In this study new approaches were examined to…’

***Response:*** *Thank you, changed. (Line 180)*

Line 142: Change to ‘from the same pool of samples from which RNA reads were retrieved…’

***Response:*** *Thank you, changed. (Lines 182)*

Line 143: Change ‘confidante’ to ‘confident’

***Response:*** *Thank you, changed. (Line 183)*

Lines 140-150: Many grammatical errors, please correct this.

***Response:*** *We have performed additional proof-reading. (Lines 180-190)*

Line 153: The term NEC was already introduced, so the abbreviation can be used.

***Response:*** *Thank you, changed. (Lines 194)*

Lines 155-164: Move to introduction section.

***Response:*** *We describe here the reasons behind our analysis of Escherichia ssp. and present the results of relative abundance calculations of the different bacterial genera. We have modified this section to include additional text about relative abundance. (Lines 195-207)*

Figure 1: Change the order of A,B,C and D, since now the authors first refer to figure 1D, and later on to A B and C.

***Response:*** *Figures were reordered according to their reference in the text.*

Line 177: The authors describe a significant difference in cydAB to cyoABCD ratio across all time points. I wonder how statistical analysis on the separate time points has been performed, since collection of more than 1 sample on each time point was rare, as shown in table 2, and statistical analysis can therefore not be performed. Maybe the authors meant to say something different, please clarify.

***Response:*** *Thank you for the comment. Authors meant across all time blocks, text was changed accordingly. (Line 231-232) All of the samples are taken in the time period before NEC is generally detected (after 30-40 days of life), and comparisons of diametric ratios between NEC and control infants were done across all samples (n=10 NEC and n=10 control), on an earlier time block (day of life 10-19, n=5 NEC and n=5 control), and on a later time block (day of life 20-37, n=5 NEC and n=5 control). This topic is discussed in the study design section under methods section.*

Line 184: Authors refer to figure 1CD, while to understand the differences between using KEGG database of infant MAGs, one should compare A with C.

***Response:*** *Thank you for highlighting this point. We changed sentences referring to the figures. Now it writes:*

*“We found that mapping RNAseq reads to genes from Escherichia spp. from each infant microbiome MAG had more significantly distinguishable DR between NEC and control infant then DR found for RNAseq reads mapped to genes retrieved from KEGG database (Fig. 1A and Fig. 1B, respectively).” (Lines 235-238)*

Lines 182-184: Although not significant, the same trend can be observed when using genes from the KEGG database. Could this be a matter of small sample size?

***Response:*** *It may be a matter of small sample size, but we cannot say at this point without additional samples.*

Lines 187-188: It could also be a matter of picking the right E. coli strain from the database. Why was E. coli K12 selected? This is a laboratory strain and may not reflect functional properties (and therefore genes) that are needed to thrive in the human gut. A better option may be to use public available whole genomes from clinical isolates from the NICU.

***Response:*** *Thank you for the comment. We added another point that generally within an individual person strains are stable (see citations in text), thus MAG’s don’t need to be sequenced and assembled from each sample metatranscriptome is sampled, if the experiment measures gut microbiome transcription in the same individuals.*

*The following text was added:” The mapping exercise shown here might potentially describe the extreme end of strain heterogeneity, as K-12 E. coli is a laboratory strain that can be different enough from gut E. coli to result in inquorate RNA read mapping. Choosing genomes from databases originating from gut microbiome databases might be more adequate for RNA mapping than KEGG database, as long as it is possible to choose genomes most similar to those genomes for which RNA was sequenced. Thus, while using genome databases is useful for taxonomic identification, as done here to confidently identify MAG’s in our samples, for accurately mapping and quantifying RNA reads it is probably most advantageous to use sample-specific genomes. It is also important to note that potentially fewer metagenomes are required per individual than meta-transcriptomes, as it was previously shown that specific strains can remain stable within an individual human host” (Lines 377-388)*

Line 193: Add full name of the genes

***Response:***  *In line 248 added “(****F****umarate and* ***n****itrate* ***r****eductase,* ***A****erobic* ***r****espiration* ***c****ontrol protein)*

Line 194: Change FNR to fnr

***Response:*** *We are addressing to the protein itself and changed the sentence to make this more clear: “the regulatory mode is through changes in FNR protein conformation at different oxygen levels” (Line 250)*

Line 202: Change to ‘hypothesized’.

***Response:*** *Thank you, changed. We didn’t mean to hypnotize anyone… (Line 258)*

[Discussion]

Line 298-299: Did the authors consider to mimic low and high oxygen exposure in an in vitro setting or animal model to confirm their method? Would rather do that to begin with than studying a bigger group of infants. Currently, the method is not validated, but directly applied to clinical samples during which too many aspects are based on assumptions.

***Response:*** *Such experiments will be valuable for validating the DR approach, as acknowledged in the discussion (Lines 364-367), but we believe these difficult experiments are outside the scope of this project.*

**Reviewer #2**:

This paper describes an interesting approach to make more (or better) use of meta-transcriptomics data. Even though a priori knowledge is needed, thus limiting it to exploration of already established pathways and transcriptional relationships.
This idea to make use of the metagenomic derived MAG's poses an interesting approach. However, the described methodology is, in the current manuscript text, not always clear enough to follow.

***Response:*** *Thank you for these comments. We have re-written the methodology section and hope it now clearer to understand (see specific responses below).*

General remarks:
- The tone of the abstract is rather strong. The main text is better nuanced, especially due to the section on the limitations (i.e. the Confounding factors section that describes the sample size, subject "baseline" differences, etc). Strongly suggest to tone down the abstract to prevent overselling.

***Response:*** *Thank you for this comment. We have toned down the abstract to describe more accurately the experimental design and confounding factors. (Line 28-38)*

- The definition and formula of the diametric ratio remain a bit vague. Though it is understandably depending on each gene, it would help to literally state the coupled genes (Table 3 does not provide direct linking). Furthermore, actual descriptive statistics on the transcript abundances of the used examples would greatly help following the method (so the A's and B's of each of the example gene sets, as the boxplots only show the final outcome ratio). Also, any given external condition/stimulus would likely affect several genes, what to do with other genes that are known to be affected by oxygen or infllammation status?

***Response:*** *Thanks for this comment. We rewrote the description of the diametric ratio in the methods section (see also our response to reviewer #1). (Lines 143-160)*

*Coupled genes are stated in the header of each section of the results, and Table 3 has been modified to clearly state which genes are coupled together and which variables they represent in equation (a).*

*To highlight the advantages of using diametric ratios, Table 4 was added. This table compares the averages and variability of transcript abundances to averages and variability of DR across time blocks. It shows how DR reduced variability across samples allowing a more reliable statistical analysis. (Lines 208-218)*

- Although reasoning to focus on E.coli is understandable, it does make the reader wonder what happened to all the other MAGs. How many were there per sample?

***Response:*** *As mentioned in the text, other MAG’s were not adequately represented in all samples to preform DR analysis. A supporting data figure has been added to show the relative abundances of different genera, and supporting data table describing the relative abundance and coverage depth for each genome has also been added as well. Between 10-24 MAG’s found in each sample, with an average of about 15 MAG’s per sample. (Lines 111-123)*

- In line with the previous point how well are the MAGs assigned to a taxonomic unit? How confident is the assignment to E.coli? In the Materials and Methods section the phylogenetic assignment appears to been done at the very high phylum level. How deep were the samples sequenced (both transcriptome and metagenome)? Especially since the rather short read HiSeq platform was used, a reasonable depth would be needed. These technical details, that are needed to assess the quality of the reported analysis, are missing or should be reported directly into the main text.

***Response:*** *Taxonomic classification was performed on the species level. If more than 50% of proteins on a scaffold shared the same taxonomic affiliation, the scaffold was designated according to that affiliation. Each of our Escherichia spp. had more than 85% of proteins sharing same affiliation. A description of this method and its results were added in the methods section (Lines 112-119).*

*As previously stated, not all infants had E.coli species, one infant had E.vulneris. So we combined the analysis of all Escherichia spp. on genus level. As mentioned in previous comment, a supporting data table showing relative abundance and coverage depth of each genome has also been added for each genome.*

- Though conceptually it could make sense, the conclusion that mapping RNA reads to sample-specific MAGs is more significantly distinguishable compared to mapping to genes retrieved from an outside database is hard to follow in the text. From the results it is not particularly clear if the mentioned E.coli focus means only one (or more) found MAG(s) that was/were assigned to E.coli was/were used or not. Assuming that this is a (set of) MAG(s) identified in these samples, then the comparison appears to be made against KEGG references genes, but these only originate from one strain (K-12 MG1655)? Is the MAG assignment considered accurate to strain level (see earlier point)? If not, then why not compare to various, if not all, E.coli genes in the online repositories? Especially for E.coli there is a huge genetic variation between all the known strains. Comparing to a single set of genes can therefore not prove this is a better method.

***Response:*** *Analysis with dRep indicated that many individual strains of E. coli were identified in infant samples. Thus, the genome strain found in each infant was used to map that infant’s RNA sequences. The comparison was eventually collated to only include sequences of the Escherichia genus, but this analysis was performed using genomes assembled from each individual infant (Lines 196-200). We do acknowledge that K-12 may not be ideal for KEGG-based analysis (as discussed on reviewer #1 comments as well).*

Text is mostly well readable though some grammar checks may bring some improvements - some specific examples:
- line 196 "higher when there are more anaerobic conditions [24].": Did you mean "higher when the condition is more anerobic [24]". Perhaps it is even better to state "less/lower oxigen".

***Response:*** *Thanks for this comment. More grammar checks were done to improve text. Text was changed to:”* *However, the transcription of arcA, which is regulated by fnr, increases when low oxygen conditions prevail [28].” (Line 251)*

- line 236 'provide" --> provides ?

***Response:*** *Thanks. Text was changed*
