# Supplementary material for: Combined analysis of microbial metagenomic and metatranscriptomic sequencing data to assess in situ physiological conditions in the premature infant gut
Source: PLoS One. 2020 Mar 4;15(3):e0229537. doi: 10.1371/journal.pone.0229537 (PMC7055874; doi:10.1371/journal.pone.0229537)
Supplement: Attachment — Submitted filename: 2nd revision Response to Review Comments.docx [file pone.0229537.s010.docx]

**2nd revision Response to Review Comments**

**Reviewer #1:**

Yonathan Sher et al made changes to the manuscript accordingly. I, however, remain hesitant about the validity of the presented approach and drawn conclusions, because of unclarities on various levels (sample type, data processing, DR criteria, data interpretation and no validation). See detailed comments below.

[General]

Is a minimum coverage depth required for the DR to be ‘accurate’? If so, what is suggested? If not, wouldn’t that be required?

**Response:** In our data set the minimum cutoff was above zero coverage depth (as it will result in extreme DR values of 1 or 0; Lines 175-176). We agree that establishment of a minimum coverage threshold would be a useful addition, but due to our small sample size we were unable to further specify a coverage threshold, as described in the text when discussing confounding factors of our dataset (Lines 413-415). Although, when we did try to apply a coverage depth cutoff of x1, we did get similar trends of microbial transcriptional response to lower oxygen levels in NEC infants.

[Abstract]

Line 21-22: Organism abundance is actually taken out of the equation. Authors don’t ‘correct’ for organism abundance, because the DR is not affected by organism abundance. I suggest to rephrase this to make it clearer to the readers.

**Response:** Thanks for the suggestion. We rephrased the sentence in the abstract to imply that we used an approach that avoids biases of organism abundance rather than correcting it (Line 21).

[Introduction]

Line 40-41: Suggestion to change to “Physiological conditions within the gut are important metrics to measure when studying gut inflammatory diseases [1,2], yet are notoriously difficult to measure in vivo.

**Response:** Thanks for the suggestion, text was changed (Line 41).

Line 66-67: Based on the analysis described herein, the conclusion that the method is widely applicable cannot be drawn.

**Response:** This text was deleted to tone down the message.

[Methods]

Line 73: Suggestion to say ‘As previously described…’ instead of ‘as noted previously…’

**Response:** Thanks for the suggestion, text was changed (Line 74).

Line 78-79: Clarify on which basis the time blocks were stratified. Sample number-driven (so each block has 5 samples) or ‘biology’-driven, or other?

**Response:** Thanks for this comment. The time blocks were created to both get a sense on the changes along time in DR between NEC and control as well as to maintain a balanced number of samples. Text was changed to clarify it (Lines 80-82).

Table 1: Did any of these infants receive breathing support? Continuous positive airway pressure can result in an increased oxygen supply to the GI tract.

**Response:** Each of the infants did receive some form of respiratory support at various time points in the study, but the details of the respiratory support (e.g. days of positive pressure ventilation) were not recorded as part of the study. Even if they were, the small sample size would not allow for any meaningful commentary on the impact of these variables. However, we agree that the impact of respiratory support (both positive pressure and also inhaled oxygen) upon oxygen tension in the distal intestine is an understudied topic that we plan to formally explore in subsequent well powered studies.

Line 110: This is just a link to the website, how can I find these particular scaffolds? Please add a accession number or something similar.

**Response:** Table 1 was added to supporting information, showing accession phrases to the different *Escherichia spp.* scaffolds used in this study. Accession phrases for scaffolds associated with different dereplicated MAG’s used in this study can be found in the ‘scaffold to bin’ files in supporting information, as indicated in previously (Lines 115-117).

Line 102: In this section, it is unclear if it is about how RNA OR DNA sequences are processed. I suppose genome reconstruction is based on metagenomics data?, but in the first sentence (Line 103) RNA is mentioned as well, as well as in the later part of this section (Lines 108-111).

**Response:** We moved all RNA sequence processing to the section describing DR analysis, to separate between RNA and DNA sequence processing (Lines 141-143).

Line 113: Same here, I assume metagenomics data is used for phylogenetic profiling, but it is important to mention it somewhere nevertheless.

**Response:** We added text indicating assembled metagenomic sequences were used for phylogenetic profiling (Lines 121-122)

Line 125: Wouldn’t you expect to obtain more MAG’s from feces? I understand these are samples from preterms in early life, and therefore the microbiota is still minimal and developing. However, looking at the relative abundance profiles I would expect to identify more species than 15. Is it a methodological issue?

**Response:** While we can't rule out the effect of systemic biases that effect all sequencing-based studies (including biases in DNA extraction and sequencing efficiencies), hospitalized premature infants are known to host extremely simple microbial communities given their isolation and frequent broad-spectrum antibiotic regimens. 15 species or less microbial species is not unexpected [1,2].

1. Gibson MK, Wang B, Ahmadi S, Burnham C-AD, Tarr PI, Warner BB, et al. Developmental dynamics of the preterm infant gut microbiota and antibiotic resistome. Nature Microbiology. 2016;1: 1–10. doi:[10.1038/nmicrobiol.2016.24](https://doi.org/10.1038/nmicrobiol.2016.24)

2. Olm MR, Brown CT, Brooks B, Firek B, Baker R, Burstein D, et al. Identical bacterial populations colonize premature infant gut, skin, and oral microbiomes and exhibit different in situ growth rates. Genome Res. 2017; gr.213256.116. doi:[10.1101/gr.213256.116](https://doi.org/10.1101/gr.213256.116)

Line 125: Could the fact that most MAGs were Escherichia be a result of availability (redundancy) in the database?

**Response:** We did not mean to say that most MAG’s were from *Escherichia* genus. We wrote MAGs from *Escherichia* were ubiquitous in most samples, that is appearing in most samples. Being an enterobacteria it makes sense it will appear in most samples. We added wording in the text to make it clearer. (Lines 214-216)

Line 133: Change to “To calculate Diametric Ratios (DR), RNA reads were…”

**Response:** Changed, thanks. (Line 142)

Table 3: The table shows different ‘relationships’ between the coupled genes for DR. Some are opposing in a specific condition (e.g. number 4 in response to NO), while some conditions are opposing which results in transcription of different genes (e.g. number 5). Please clarify on which terms genes are considered opposite transcriptional responses. With other words, which criteria need to be fulfilled for genes to be identified as ‘opposite transcriptional responses’. For example, why are arcA and cyoABCD no opposing genes, as one goes up in anaerobic conditions, and the other goes up in aerobic conditions (which is a similar difference in condition as number 5 for osmolarity).

**Response:** Thank you for this comment. We designate coupled genes for DR genes that their transcriptional response is opposite to a given physiological condition or respond to opposing physiological condition. We added this definition in the text (Line 164-167).

According to this definition *arcA* and *cyoABCD* can be designated as coupled genes for DR calculations. We went along and calculated DR between these genes, where *arcA* is the α component and *cyoABCD* are the β component. We got similar results as the other coupled genes for DR genes responding to oxygen, with significantly higher DR in NEC infants compared to control infants (welch’s ttest p<0.01).

Line 173-174: Please add the version of R and the ggplot2 package.

**Response:** R and the ggplot2 package versions were added.

Line 175-178: I remain unsure about which comparisons are made. Control versus NEC at time block one; Control versus NEC at time block two; and control versus NEC of all samples? I am confused because the authors write “within time block”, which I interpret as comparing samples within a time block with each other, instead of comparing all samples in a time block between control and NEC.

**Response:** Thank you for this comment. We changed the text to clarify that we are comparing all samples within each time block between control and NEC. (Lines 189-194)

[Results]

Line 181: ‘a new approach was examined to..’

**Response:** Thanks, text was changed (Line 197).

Line 182-184: How can this be an approach by itself? An approach to achieve what exactly? Which potential bias are circumvented by this? In the introduction, the authors focus on one new method: the diametric ratio. To my opinion, mapping of RNA reads to MAG’s is just part of the process to calculate the DR. However, two methods for mapping are examined: mapping to MAGs and mapping to the genome of E.coli K-12 from database. I suggest to focus on the DR approach and the comparison of the two mapping methods.

**Response:** Thanks, modified the text to describe an approach composed of mapping and DR calculations. (Lines 197-204)

193: ‘to assess this approach’

**Response:** Thanks, text was changed (Line 211).

Line 196: When is a genus considered adequately ubiquitous?

**Response:** When representatives of a genus occur in most of the samples, allowing statistical analysis between NEC and control (Lines 215-216).

Line 203: The fecal microbiota does not represent the microbiota lining the mucosal layer, which may be more affected by reduced regional blood flow than luminal microbiota. In addition, the gut is not oxygen free in early life, which is important to take into consideration, particularly for the early timepoint samples (~first month, probably longer in preterm infants).

**Response:** Microbiota lining the mucosal layer may be more affected by reduced regional blood flow than luminal microbiota, but apart from surgery there is almost no way to retrieve mucosal layer microbes. In addition, differences in oxygen level can extend beyond mucosal layer into the gut lumen [1], and gut microbe are suggested to be affected by oxygen levels there [2]. As we note in the discussion, early life gut microbiomes are exposed to higher oxygen levels than later in life (Lines 452-454), and we argue that our results suggest that gut microbiomes of preterm neonates developing NEC are exposed to lower levels of oxygen.

1. Albenberg L, Esipova TV, Judge CP, Bittinger K, Chen J, Laughlin A, et al. Correlation Between Intraluminal Oxygen Gradient and Radial Partitioning of Intestinal Microbiota. Gastroenterology. 2014;147: 1055-1063.e8. doi:[10.1053/j.gastro.2014.07.020](https://doi.org/10.1053/j.gastro.2014.07.020)

2. Rivera-Chávez F, Lopez CA, Bäumler AJ. Oxygen as a driver of gut dysbiosis. Free Radical Biology and Medicine. 2017;105: 93–101. doi:[10.1016/j.freeradbiomed.2016.09.022](https://doi.org/10.1016/j.freeradbiomed.2016.09.022)

Line 211-212: ‘Transcript abundances of each of these two sets of genes (either cydAB or
212 cyoABCD, without any normalization accounting for genome abundance) was extremely variable’. Variable between what? Between samples, between NEC/control?

**Response:** Thanks for this comment. Text was added to clarify it: ”within all-time blocks of either NEC or control infants” (Line 241)

Line 214: Change to (between 93-174) – 93 instead of 92.

**Response:** Thanks, text was changed (Line 243).

Table 4: Please add the DR for all gene sets as supplementary data.

**Response:** DR for all gene sets were added as supplementary information excel file: S7_Diametric_ratios.xlsx

Table 4: Average coverage of the genes in control infants was only 2-3. Getting back to my earlier comment: “Is a minimum coverage depth required for the DR to be ‘accurate’?”

**Response:** Please see our response to the original comment.

Line 233-236: Escherichia spp were of much lower abundance in the control than NEC infants (Fig S1). May it be that Escherichia of low abundance express a different set of genes than dominant Escherichia, simply as a result of their abundance? Overall metabolic activity may, independent of oxygen levels, already be different. It looks like the gut environment of preterms developing NEC favors conditions for Escherichia growth and replication. Could a more competitive environment for Escherichia (like in control infants) alter expression of cydAB cyoABCD genes, since other activities may be of more importance for survival? Can the authors check for activity status of Escherichia, maybe by checking the expression of housekeeping genes, or by RNA coverage over the entire genome? Long story short: How sure are the authors that the difference in DR represents differences in oxygen levels at the epithelial lining of the gut, and not overall deprived activity in case of low abundance?

**Response:** Diametric ratios were designed to overcome comparisons difficulties due to differences in organism abundances (see table 4). In addition, we also examined other genes related to oxygen levels (*nrd*’s and *arcA/fnr* genes) which show similar trends to *cydAB cyoABCD* genes. While it is conceivable that the difference in these genes’ expression patterns are due to some other physiological responses, we believe that the reaction to oxygen levels is the most parsimonious explanation.

Line 258-260: Since this must be a result of RNA mapping efficiency, can the authors give an impression of the difference in gene sequence (of the specific genes of interest) between the MAGs and E coli K12?

**Response:** Thank for this comment. To get an impression of the difference in gene sequence we added a file with an alignment of MAG’s *cydA* to kegg *cydA*. Each MAG’s *cydA* had different number of mismatches (S15 txt file: CydA-Alignments.txt), ranging from 1 to 19 in *E.coli* species and 269 mismatches with *E.vulneris* *cydA*. (Lines 291-294).

[Discussion]

Line 330-331: On which criteria is the conclusion of ‘effectively used’ drawn? When is a method effective?

**Response:** We concluded that this approach is effectively used according to the data we show. The low variation of the data of DR compared to sequence abundance without DR calculation (Table 4), and significant results when mapping to MAG’s (Figure 1). We added reference to the table and figure (Line 365).

Line 412: affecting

**Response:** Thank you, text was changed. (Line 447)

Line 417-419: Would it be appropriate to screen the databases for Escherichia spp that result in the highest transcript abundance, and use this/these for analysis? In addition, would it be appropriate if the ‘best pick’ from the database is difference for each sample?

**Response:** Thank you for this question. Such an approach might be useful for choosing genomes from databases when MAG’s are not at hand. However, stringent mapping is needed to accurately quantify transcript abundance, to find ‘best hit’ genomes with highest transcript abundance.

‘best hit’ genomes will probably be different between samples from different infants, however, within an infant same strain can remain stable for long periods of time, as we discussed in the text (Lines 459-461).

Line 419-422: Rebuild sentence.

**Response:** We rebuilt and divided into two sentences: “Genome databases are valuable for taxonomic identification, as done here for confident identification of MAG’s in our samples. However, based on propositions put forth in this study, further research might further enforce the idea that MAG’s enable more accurate mapping and promote better quantification of RNA reads.” (Lines 454-459)

Line 429: Suggestion to remove “and potentially help identify biomarkers for early detection of NEC progression in premature infants.”

**Response:** Thanks for the suggestion. We agree, the text has been removed.

**Reviewer #2:**

The authors have greatly increased readability/transparency of the analysis. Whether or not this approach is practically suitable in preterm NEC setting is up to debate, but the approach to make use of metatranscriptomics in the described method is interesing and now much clearer for other parties to repeat. Weaknesses and assumptions are now much more transparent.

Minor comment(s):
- Lines 107-108 "One genome of each 107 identified bacterial species was manually chosen from each infant gut microbiome for downstream RNA analysis" --> I understand the manual choice here, but please clarify where this information (which bacterial species chosen for which smaples) can be found. Online? SI Table 1?

**Response:** The MAG’s chosen for each infant and their corresponding scaffolds can be found in the ‘scaffold to bin’ files in supporting information (Lines 115-117).

- In the Materials and Methods sections some "results" are presented among the methodology (such as the sentences in line 118-121; line 124-125). These would fit better in the Result section.

**Response:** Thank you for this suggestion- this section has been moved to the “results” section (Lines 218-226).
