# Supplementary material for: Combined analysis of microbial metagenomic and metatranscriptomic sequencing data to assess in situ physiological conditions in the premature infant gut
Source: PLoS One. 2020 Mar 4;15(3):e0229537. doi: 10.1371/journal.pone.0229537 (PMC7055874; doi:10.1371/journal.pone.0229537)
Supplement: Attachment — Submitted filename: 3rd revision Response to Review Comments_c.docx [file pone.0229537.s011.docx]

Response to 3^rd^ Review Comments

Reviewer #1: Yonathan Sher et al made changes to the manuscript accordingly, including clarification of applied data processing methods and DR criteria. I remain hesitant about whether the analysis can be repeated by another party (due to the arbitrary nature of the approach) and about its applicability in clinical setting. However, the proposed method may inspire other researchers to think in this direction, and to be aware of potential biases, when analysing their -omics data.

**Response:** Thank you for this comment, and for all of your critical comments throughout this review process. We also believe the DR approach may inspire and help future meta-transcriptomic studies.

Minor comments:
- Check grammar and spelling once more, carefully, throughout. E.g. lines 197-204: Mixed use of present and past tense (was examined, involves, were retrieved etc), same accounts for lines 214-218 (focused, was, occur, were not).

**Response:** Grammar and spelling were checked again across all the MS, specifically for coherent use of tenses.

- Based on the analysis described herein, the conclusion that the method is widely applicable
cannot be drawn (the sentence stating this has already been removed in the introduction section, but not yet in the abstract).

**Response:** Text in the abstract was changed: “Nevertheless, together with benchmarking analyses, we show here that the diametric ratio approach can be applied for evaluating the physiological conditions experienced by microbes *in situ*.”

- I suggest, for the conclusion, to focus on the approach (like lines 443-446), and not so much on the preterms/NEC. E.g. In conclusion, we show here how meta-transcriptomic data combined with sample-specific MAG’s can be applied effectively to probe the physiological conditions that gut microbial communities experience by comparing diametric ratios. Further application of this approach can bring new insights on microbe-host interactions within the GI tract systems, and potentially help identify biomarkers for disease onset/progression.

**Response:** Text was changed to focus more on approach with smaller emphasis on NEC:” Further application of this approach can bring new insights on microbe-host interactions within the GI tract systems, and potentially help identify biomarkers for early detection gut diseases, such as NEC, onset and progression.”

Reviewer #2: The Manuscript has been substantially improved. Validation of the approach remains a weaker point but this is much more addressed and the manuscript invites others to further explore and validate.

**Response:** Thank you for your comments. We also hope future studies would strengthen the validation of the DR approach.

Some minor items to consider:

DR definition:
- The definition is based on the "opposite transcriptional response" of genes to a given environmental condition. Should this be strictly kept at transcription? Or could the "opposite" term also be "different"? Because the fnr/arcA example describes that arcA react to the condition, but the fnr transcription is (expected to be) constant so fnr transcription does not rely on the environmental condition (it's translated protein FNR does react to the condition). All other gene sets are obviously of a much more "opposite" nature.

**Response:** Thank for the interesting comment. We currently rather stick to *opposite* rather than *different*, with the fnr/arcA example being an exception. We agree that further investigations of other cases should be examined to precisely define sets of genes that can be involved in DR analyses.

Methodology - suggestion to enhance repeatability of procedure by others
- in the Results (line 208-210) it is stated that RNA reads were mapped on the MAGs per sample, but later this was combined per genus level. To ensure others can repeat your approach you should state how this "combining" was done. Are mapped results/data further "aggregated" based on the gene name/annotation of the different Escherichia species/MAGs?
Perhaps this is evident from the SI files, but it would make it conceptually easier to understand for a larger audience.

**Response:** Thanks for this comment. Text was changed so it will be clearer how analysis was done on the genus level. Line 209-213

“It is important to note, that RNA reads from each sample were mapped to the *Escherichia* sp*.* genome found in the same sample, as not all of the infants had *E.coli* species. Subsequent analyses were carried out on the genus level of *Escherichia*. Therefore, calculated DRs for all *Escherichia* spp. found in the different samples were gathered and compared between NEC infants and control infants.”

Suggestion to strengthen discussion in "Confounding factors" section
- the paragraph with lines 402-412 states that the presented findings are opposite to published findings related to gestational age. Though I agree that this intuitively might seem to be the case: the figures of the DRs do not seem to show a time development in either Control or NEC group, except for norVW/norV DR but this indeed seems to go in the opposite direction. However, as the breathing support is not (properly) recorded and this might have an impact. Moreover, DR findings are "relative" and no true "later gestational age / confirmed anaerobic" infant fecal sample is analyzed and such a validation could give more insight.

**Response:** Thank you for this suggestion. The text referring to gestational age is addressed to the comparison between NEC and control samples in this specific dataset, where NEC samples were retrieved from infants at a younger gestational age (2-3 weeks younger, Table 1). This dataset is not exclusively designated to address questions regarding chronological changes in preterm infants’ gut microbiome transcriptional patterns. We agree more studies should address these questions, and we added the following text:

“..our results show that lower oxygen exposure occurred at earlier gestational age, in infants that develop NEC (Table 1; Fig. 1A; Fig. 2AB). Although, larger sized and higher time point resolution gut microbiome meta-transcriptomic studies would be fundamental to confirm shifts in aerobicity state in the intestine of premature infants.”

Some minor language items I noticed:
- line 40 "in vivo" should be in italics

**Response:** changed to italics

- I believe "spp." should not be in italics (many times throughout the text and figure legends)

**Response:** Italics **“***spp.*” was changed to regular “spp.”

- suggestion to add the word "to" (or "with") in line 405 after "compared"

**Response:** changed to “to”.

- line 407: "occur" should be "occurs"?

**Response:** changed to past tense, to “occurred”, per reviewer #1 suggestion.
